# The drug cocktail network

**DOI:** 10.1186/1752-0509-6-S1-S5

**Published:** 2012-07-16

**Authors:** Ke-Jia Xu, Jiangning Song, Xing-Ming Zhao

**Affiliations:** 1 Department of Mathematics, Shanghai University, Shanghai 200444, China; 2 Institute of Systems Biology, Shanghai University, Shanghai 200444, China; 3 National Engineering Laboratory for Industrial Enzymes and Key Laboratory of Systems Microbial Biotechnology, Tianjin Institute of Industrial Biotechnology, Chinese Academy of Sciences, Tianjin 300308, China; 4 Department of Biochemistry and Molecular Biology, Faculty of Medicine, Monash University, Melbourne, VIC 3800, Australia

## Abstract

**Background:**

Combination of different agents is widely used in clinic to combat complex diseases with improved therapy and reduced side effects. However, the identification of effective drug combinations remains a challenging task due to the huge number of possible combinations among candidate drugs that makes it impractical to screen putative combinations.

**Results:**

In this work, we construct a 'drug cocktail network' using all the known effective drug combinations extracted from the Drug Combination Database (DCDB), and propose a network-based approach to investigate drug combinations. Our results show that the agents in an effective combination tend to have more similar therapeutic effects and share more interaction partners. Based on our observations, we further develop a statistical approach termed as DCPred (Drug Combination Predictor) to predict possible drug combinations by exploiting the topological features of the drug cocktail network. Validating on the known drug combinations, DCPred achieves the overall AUC (Area Under the receiver operating characteristic Curve) score of 0.92, indicating the predictive power of our proposed approach.

**Conclusions:**

The drug cocktail network constructed in this work provides useful insights into the underlying rules of effective drug combinations and offer important clues to accelerate the future discovery of new drug combinations.

## Background

Drug combination is the combination of different agents that can achieve better efficacy with less side effects compared to its single components. Recently, it is becoming a popular and promising strategy to new drug discovery, especially for treating complex diseases, e.g. cancer [1-3]. For example, Moduretic is the combination of Amiloride and Hydrochlorothiazide, which is an approved combination used to treat patients with hypertension [4,5]. Chan *et al. *identified a combination drug, namely Tri-Luma, for combating melasma (dark skin patches) of the face based on efficacy and safety experiments [6]. Agrawal *et al. *found two effective combinatorial drug regimens to treat Huntington disease based on prescreening in *Drosophila *[7]. In addition, through the synergistic antiangiogenic effects, very low-dose combinatorial use of vinblastine (VBL) and rapamycin (RAP) was demonstrated to inhibit the proliferation of the endothelial cells much more effectively than single drug treatment both *in vitro *and *in vivo *[8]. Recently, Lehar *et al. *found that synergistic drug combinations may have less side effects, because synergistic drug combinations are generally more selective to particular cellular contexts than single agents, and the dosage of each compound in combination will be reduced comparatively [9]. Despite of the extensive efforts that have been made to discover new drug combinations in the past few decades, the majority of effective combinatorial drugs used in clinic were discovered through experiences, which generally require labor-intensive and time-consuming "brute force" screening of all possible combinations among the approved individual drugs [10]. In a drug combination, a drug may promote or suppress the effect of another one. For instance, cyclosporine increases the effect of sirolimus, while bupropion decreases the effect of cyclosporine. As a result, two drugs may have a totally new effect that is different from the ones of either individual drugs [11,12]. Accordingly, the presence of potential drug-drug interactions (DDIs) and the possibility of pharmacokinetic interventions between the drugs could confound the identification of effective drug combinations [13]. Furthermore, the number of possible combinations will increase exponentially with the increasing availability of single drugs. For example, in the case of four drugs, there will be six possible combinations. This number would be enormous considering the fact that there are thousands of approved drugs. Due to the huge search space of possible combinations between known drugs, the identification of optimal and effective drug combinations is a non-trivial and challenging task.

Therefore, it is necessary to develop effective *in silico *methods that are capable of discovering new drug combinations prior to combination synthesis and practical test in the lab. Owing to the completion of human genome sequencing projects and the advancement of molecular medicine, extensive system biology efforts have been made to discover new combinations based on molecular interaction networks [14,15] in the past few years [16-19]. Nevertheless, there is still a long way to go before we reach the stage of devising generally applicable and effective prediction models. Recently, there have been considerable progresses in developing new approaches for identifying drug-drug interactions and even drug combinations [13]. In this context, Geva-Zatorsky *et al. *have recently found that the protein dynamics in response to drug combination can be accurately described by a linear superposition of the dynamics under the corresponding individual drugs [16]. Their study indicated that protein dynamics of three- and four-drug combinations can be predicted based on the drug combination pairs, thereby providing a useful way for reducing the search space of possible drug combinations. Calzolari *et al. *devised an efficient search algorithm originated from information theory for optimization of drug combinations based on the sequential decoding algorithms [17]. More recently, researchers have also developed computational frameworks for predicting drug combinations and synergistic effects based on high-throughput data [18-20].

In this work, we study the drug combinations in terms of their therapeutic similarity and the network topology of a drug cocktail network constructed from the effective drug combinations deposited in the Drug Combination Database (DCDB) [21]. We find that the drugs in an effective combination tend to have more similar therapeutic effects and share more interaction partners in the context of drug cocktail network. We further develop a statistical approach called DCPred to predict possible drug combinations and validate this approach based on a benchmark dataset with all the known effective drug combinations. As a result, DCPred achieves the overall best AUC (Area Under the receiver operating characteristic Curve) score of 0.92, demonstrating the predictive capability of the proposed approach and its potential value in identifying new possible drug combinations.

## Results and discussion

### The drug cocktail network

**Figure 1:**
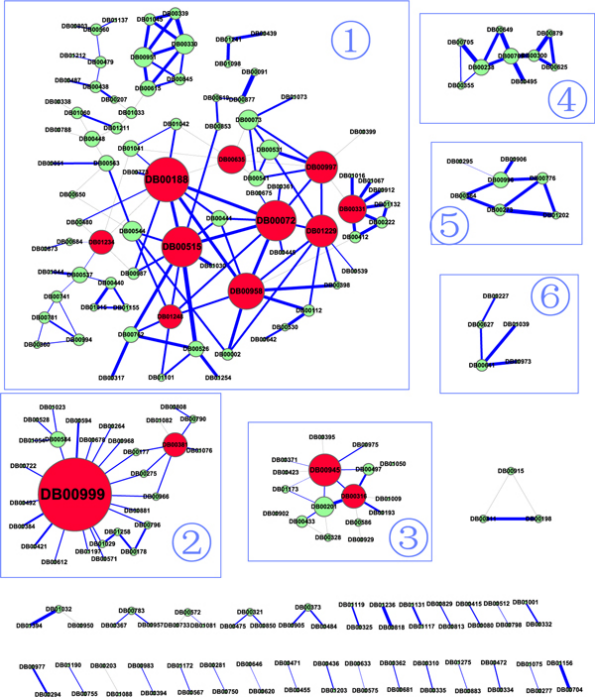
**The drug cocktail network**. A node represents a drug and an edge denotes an effective combination consisting of the two drugs linked by the edge. The hub drugs that have more than 6 neighbors are colored in red. The size of each node approximates its degree, the width of each edge approximates the therapeutic similarity (see equation 3) between the two drugs linked by the edge, and a grey edge means that the two drugs linked by that edge have completely different therapeutic effects. The numbers in panel 1-6 represent the top six largest child networks from the drug cocktail network.

In this study, we extracted 239 known effective pairwise drug combinations from DCDB [21]. The information of ATC code for each drug was obtained from DrugBank [22]. Based on these datasets, we constructed a drug cocktail network with 215 nodes and 239 edges (see Figure 1 for the visualization of this network), where nodes represent the drugs and an edge is connected if two drugs are found in an effective drug combination. Building up this network can thus give the readers a visual impression of the relationships between drugs that can form effective combinations. Moreover, the network theory can be utilized to explore possible combinatorial mechanisms between drugs. In Figure 1, the size of each node approximates its degree, and the width of each edge approximates the therapeutic similarity (*TS*) (as defined in Equation 3) between the two drugs linked by the edge, while the grey edges indicate that the two drugs linked by the edge have totally different therapeutical effects. In addition, we found 102 drugs that have at least two neighbors in the drug cocktail network, which we termed as "star drugs" hereafter and 91 of which have target protein annotations in DrugBank.

**Figure 2:**
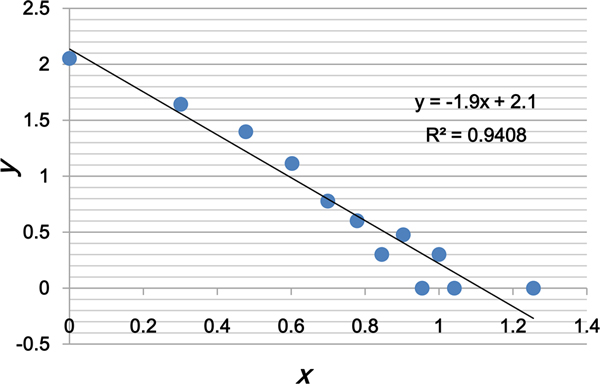
**The degree distribution of the drug cocktail network**. The *x*-axis represents the common logarithm of the value of degree *k*, while the *y-*axis represents the common logarithm of the fraction of drugs that have the degree of *k*.

Since most of biological networks are scale-free networks [23], we analyzed the topology of the drug cocktail network in order to find out whether it is also a scale-free network. The degree distribution of the drug cocktail network is shown in Figure 2. It is evident that the degree distribution follows a power law distribution, suggesting that it is indeed a scale-free network. That is, the fraction *P(x) *of nodes in the drug cocktail network having *x *connections to other nodes can be described as:

(1) $$p\left(x\right)\propto cx^{-\alpha }$$

where *c *= 2.1 and *α *= 1.9 in this case.

As the drug cocktail network shown in Figure 1 is not fully connected, the top 6 largest subnetworks were chosen for further analysis. We considered the drug cocktail network as the union of these 6 subnetworks hereafter unless stated specifically. In particular, each subnetwork was found to be enriched for one or several therapeutic classes according to the ATC classification system, as shown in Table 1. In other words, the drugs having similar therapeutic effects tend to be clustered together in the drug cocktail network.

**Table 1:** The enriched ATC codes for child networks

Subnetwork	Number of drugs	Enriched ATC codes: Frequency
1	84	L:40, J:24, A:16, S:11
2	29	C:28
3	17	N:8, M:7
4	9	J:9
5	7	N:7
6	5	J:5

The enriched therapeutic effects represented by the ATC codes (first level) for the top six largest child networks, where the numbering for each child network is consistent with that shown in Figure 1. Here, the enriched ATC codes mean that they occur more frequently, either more than 10 times or accounting for more than 40% of all ATC codes assigned to the drugs in the child networks.

To test our hypothesis that the drugs in one combination tend to have similar therapeutic effects, the drug cocktail network was compared against random combination networks. For this purpose, a therapeutic similarity (*TS*) score was calculated for each drug pair, and the average of all *TS *scores was used as the *TS *score for the whole drug cocktail network. The random combination networks were generated by randomly shuffling the edges while still preserving the degree for each node [24] in the drug cocktail network. This procedure was repeated for 1,000 times. To examine the statistical significance of the difference between the drug cocktail network and random combination networks, one *P*-value was calculated as the ratio that the *TSs *of random combination networks are larger than that of the drug cocktail network during the 1000 randomizations. The results are shown in Table 2 at different ATC code levels ranging from 1 to 4. The calculated *P*-values of the drug cocktail network across ATC code levels 1-4 are all equal to 0, strongly suggesting that the real drug combinations significantly differ from the random combination networks. Note that the 5^th ^ATC code level was not considered here, as there is only one drug combination having identical ATC codes for all the five levels in the drug cocktail network. This means that the 5^th ^ATC code level is not suitable for performing statistical analysis and thus it is not included in the analysis.

**Table 2:** The comparisons between drug cocktail network and random networks

ATC code level	1	2	3	4
*P*-value	0/1000	0/1000	0/1000	0/1000

The *P*-value at which the ratio of the therapeutic similarity (*TS*) score of a random network is larger than that of the drug cocktail network in the randomization tests of 1000 times at different ATC code levels.

**Figure 3:**
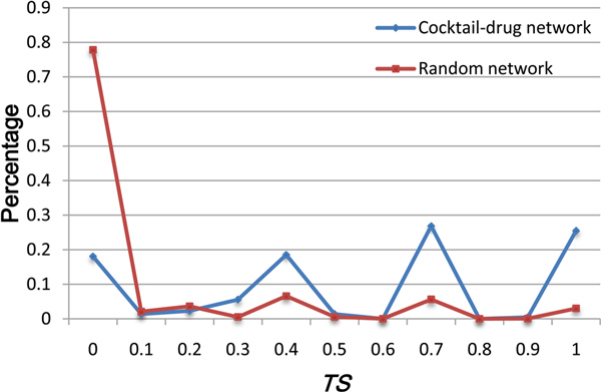
**The distribution of the *TS *scores between star drugs and their neighbors**. Blue and red lines represent the drug cocktail network and random network, respectively.

Furthermore, we studied the therapeutic effects for the "star drugs" and their neighbors in the drug cocktail network in order to reveal whether the star drugs have therapeutic similarities to all their neighbors. Figure 3 shows the distribution of the *TS *scores for star drugs and their neighbors. For the effective combination pairs involving star drugs, 82% have therapeutic similarity, and most of the star drugs have similar therapeutic effects as the majority of their neighbors. In contrast, 78% of the combination pairs in the random network do not have any therapeutic similarity. These results suggest that one star drug tends to be used in combination with drugs that have similar therapeutic effects as the star drug.

**Figure 4:**
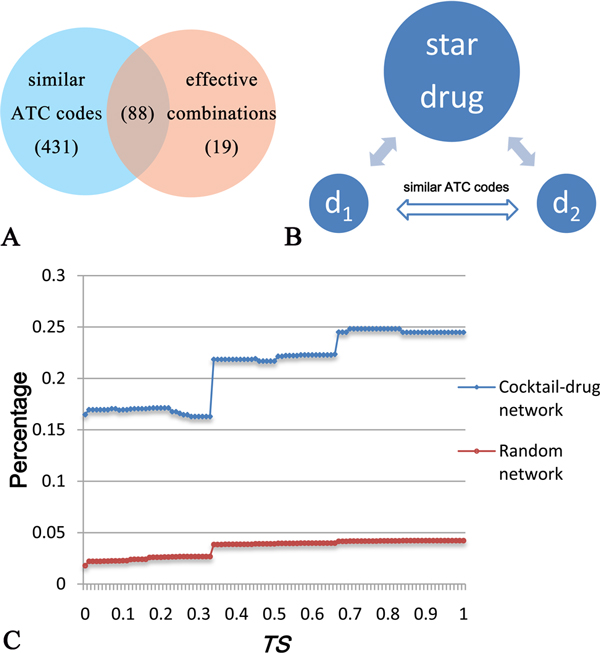
**Star drugs and their neighbors**. (A) The distribution of neighbor drug pairs of star drugs. The neighbor pairs of star drugs can be classified into two groups, according to whether they have similar ATC codes, or whether they are used as effective combinations. (B) Schematic view of the relationship between two neighbors *d_1 _*and *d_2 _*of a star drug. (C) The percentage of effective combinations within neighbor drug pairs with *TS *equal to or larger than a certain threshold. Blue and red lines represent the drug cocktail network and the average of 1000 randomly generated combination networks, respectively.

Moreover, we also investigated the distribution of neighbor drug pairs of star drugs (Figure 4A and 4B), attempting to answer whether or not the drug pairs that share a star drug have therapeutic similarity. To address this, we divided the neighbor drug pairs of a star drug into two groups, according to whether they have similar ATC codes, or whether they are approved effective combinations. We then calculated the percentage of effective combinations among drug pairs that share a star drug and have a *TS *score equal to or larger than a certain threshold (Figure 4C). From Figure 4C, we can see that the more similar therapeutic effects (as reflected by the *TS *score) two drugs have, the more likely they are effective combinations. Another important observation is that the combinations between drugs sharing similar therapeutic effects and star drugs are more likely effective combinations.

In various networks, the hub nodes are generally considered to play important roles [25]. Therefore, we next studied the 14 hub drugs in the drug cocktail network, all of which have more than 6 neighbor drugs. The largest two hub drugs are DB00999 (Hydrochlorothiazide) and DB00072 (Trastuzumab). Hydrochlorothiazide is used to treat high blood pressure and edema [26,27]. According to the annotations in DrugBank and DCDB, we found that all the 18 drug neighbors of hydrochlorothiazide can be used to cure hypertension while all the drug combinations involving hydrochlorothiazide have been used to treat hypertension. Among these 18 combinations, 11 combinatorial drugs target different but related pathways while the other 7 ones target unrelated pathways (Additional file 1). In the case of Trastuzumab used to treat HER2-positive metatsatic breast cancer [28,29], 5 of its 10 neighbor drugs are used to treat breast cancer, while the other 5 have pesticide effects on neoplasm or other cancers. All the 10 drug combinations are used to treat breast cancer except the one used for treating gastric cancer. Additionally, 8 drug combinations target related pathways, while the other two target different unrelated pathways or cross-talking pathways (Additional file 2). Finally, these results, together with the consistent findings shown in Figure 3, strongly indicate that star drugs tend to have similar therapeutic characteristics as their neighbors.

**Figure 5:**
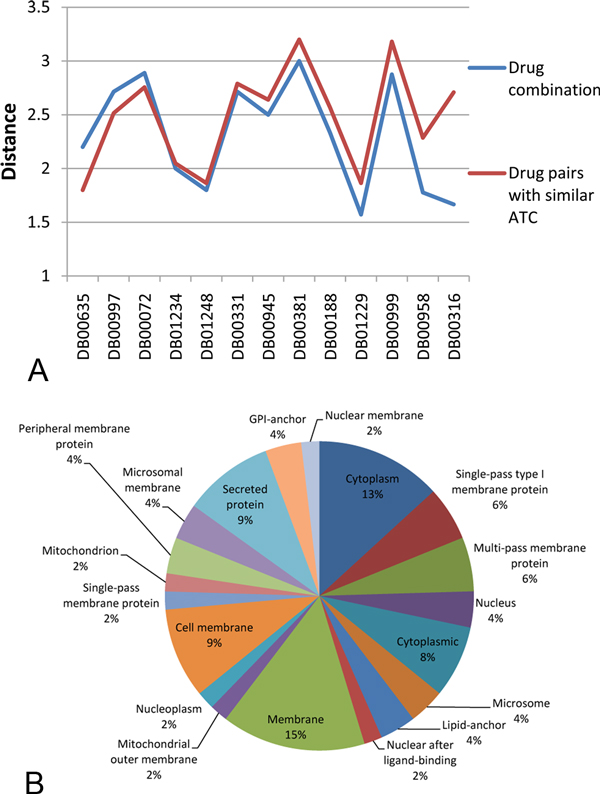
**Target proteins of hub drugs**. (A) The blue line represents the shortest distances between the targets of hub drugs and the targets of their combination partners, while the red line represents the shortest distances between the targets of hub drugs and the targets of drugs that are therapeutically similar to hub drugs. (B) The distribution of cellular localizations of the target proteins of hub drugs.

In addition, we investigated the proteins targeted by the 13 hub drugs in the drug cocktail network that have target information. By mapping all proteins targeted by the drugs in the drug cocktail network to the human protein-protein interaction network retrieved from STRING database [30], we found that, in terms of the shortest distance between target proteins, hub drugs tend to have a closer relationship with their combination partners than the drugs having similar ATC codes (see Figure 5A). Furthermore, we analyzed the cellular localizations of these target proteins of the 13 hub drugs(see Figure 5B). More than 70% of the target proteins of the hub drugs are membrane proteins, which is reasonable considering that membrane proteins are widely involved in various biological processes and represent the largest class of drug targets.

### Implication of drug cocktail network for possible drug combinations

As shown in Figure 3, 82% of the combinations between star drugs and their neighbors have therapeutic similarity, and most of the star drugs have therapeutic similarity to the majority of their neighbors in the drug cocktail network. Additionally, most of the effective combinations are observed to be located in the vicinity of drug pairs with similar ATC codes. Hence, it is possible to predict drug combinations from the set of drug pairs with similar ATC codes. Nonetheless, we found that there are only 74 known effective combinations in all of the 1181 possible combinations with similar ATC codes. Since the number of effective drug combinations is considerably smaller than that of random combinations between drugs having similar ATC codes, it is a challenging but crucial task to discover the effective combinations from the pool with a vast number of random combinations.

**Figure 6:**
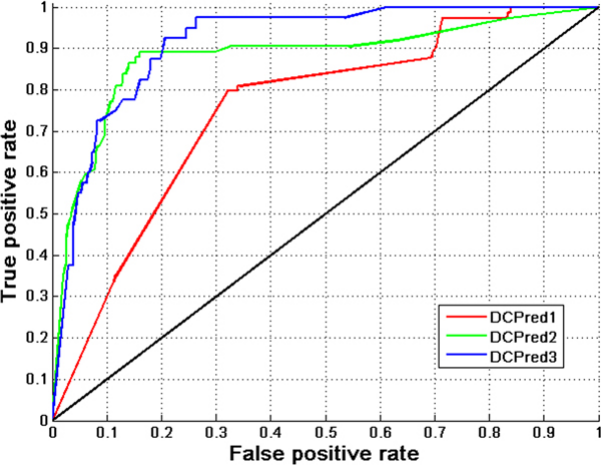
**The ROC curves of different DCPred models**. DCPred1 uses *TS *only, DCPred2 uses *TS *and drugs with at least 2 neighbors, while DCPred3 uses *TS *and drugs with at least 3 neighbors, respectively.

In Figure 4B and 4C, we can see that if two drugs with similar ATC codes have a common neighbor in the drug cocktail network, they are more likely to be combined together. Therefore, we assume that the two drugs having similar ATC codes and sharing a significantly larger number of common partners in the drug cocktail network are more likely to be combined effectively. Based on this assumption, we further developed a new statistical approach called DCPred to test this hypothesis and applied it to predict and rank all the possible drug combinations (See Materials and methods for more details). In particular, three different versions of DCPred were considered in this work, including DCPred1 considering *TS *only, DCPred2 considering *TS *and drugs with at least 2 neighbors, and DCPred3 considering *TS *and drugs with at least 3 neighbors. In the case of DCPred2 and DCPred3, all possible drug combinations were ranked in ascending order according to the *p*-value by equation (4), and the top ones were considered as putative effective drug combinations. While in the case of DCPred1, all possible drug combinations were ranked in descending order according to the *TS *value by equation (3), and the top ones were considered as putative effective drug combinations. The ranking list of drug combinations can be found in the additional files (Additional file 3 and 4). We found that two drugs with more common neighbors generally have higher rankings. Using the set of 74 effective combinations as the gold standard while the 1107 random ones as negative set (Additional file 3), we evaluated our approach in identifying new drug combinations. Figure 6 shows the ROC curves [31] obtained by different methods, where the drug pairs ranked above a given threshold were predicted as effective drug combinations (positives), while the rest were regarded as negatives. We then calculated the area under the ROC curves (AUC) [32] for these different DCPred models. As a result, DCPred2 achieved an AUC score of 0.88 (the green curve in Figure 6), in comparison with the AUC of 0.75 for the *TS*-based method (DCPred1) (the red curve in Figure 6). To comprehensively evaluate the predictive power of the three models, we also calculated three other performance indexes: Sensitivity, Specificity and Accuracy at varying thresholds for DCPred1, DCPred2 and DCPred3 models (See the Additional file 5, 6 and 7, respectively).

Of the top 35 ranked drug combinations inferred by our models, 63% of them (22/35) are known effective drug combinations according to DCDB, and 37% (13/35) do not have any annotations in DCDB (Table 3). Nevertheless, 4 out of these 13 drug combinations were reported in the literature, i.e. the 13^th^, 22^th^, 34^th ^and 35^th ^in the ranking list (Table 3). The 34^th ^ranked one is a combination of irinotecan and capecitabine, known as XELIRI, and used to treat metastatic colorectal cancer [33]. Alfonso *et al. *demonstrated that XELIRI is effective and safe as the first-line chemotherapy for treating advanced colorectal cancer or metastatic colorectal cancer [34]. The 13^th ^ranked one is the combination of docetaxel and gemcitabine, the former interferes with the normal function of microtubule growth and destroys the cell's ability to use its cytoskeleton in a flexible manner, while the latter inhibits thymidylate synthetase leading to inhibition of DNA synthesis and cell death [35,36]. Levy *et al. *found that gemcitabine-docetaxel combination has a favorable risk-benefit profile and is an important new treatment option for women with metastatic breast cancer [37]. The 22^th ^one is the combination of sorafenib and bevacizumab. The former interacts with multiple intracellular (CRAF, BRAF and mutant BRAF) and cell surface kinases (KIT, FLT-3, VEGFR-2, VEGFR-3, and PDGFR-ß) to reduce blood flow to the tumor for the treatment of patients with advanced renal cell carcinoma [38], while the latter binds VEGF and prevents the interaction of VEGF to its receptors (Flt-1 and KDR) on the surface of endothelial cells [39]. Consequently, this prevents blood vessel proliferation and tumor metastasis for metastatic colorectal cancer and HER2-negative metastatic breast cancer. Azad *et al. *demonstrated that complementary inhibition of VEGF signaling has synergistic therapeutic effects, and this combination therapy has promising clinical activity over ovarian cancer [40]. The 35^th ^one is the combination of thalidomide and lenalidomide. Thalidomide has been successfully introduced to treat multiple myeloma and its analogue, lenalidomide, is also effective in relapsed refractory myeloma [41]. The Thalidomide-lenalidomide combination can induce tumour cell apoptosis directly or indirectly by altering bone marrow microenvironment, and can be used in combination to treat multiple myeloma [42]. Both drugs bind to a common target PTGS2, which may play a role as a major mediator of inflammation and/or a role for prostanoid signaling in activity-dependent plasticity [43]. Thalidomide and lenalidomide have been shown to significantly improve the overall and disease-free survival. Combination of these two drugs has recently emerged as a promising combination strategy to improve the patient outcome and drug toxicity, especially in the treatment of multiple myeloma (MM) and hematologic cancers [44].

**Table 3:** The novel predictions of DCPred2

Rank	Drug components	*P*-value	Common targets	Reported effective combinations?
10	DB00275; DB00177 Olmesartan; Valsartan	4.35E-05	AGTR1	No
11	DB00275; DB00966 Olmesartan; Telmisartan	4.35E-05	AGTR1	No
12	DB00177; DB00966 Valsartan; Telmisartan	4.35E-05	AGTR1	No
13	DB01248; DB00441 Docetaxel; Gemcitabine	4.83E-05	None	Yes
14	DB00526; DB01248 Oxaliplatin; Docetaxel	4.83E-05	None	No
22	DB00398; DB00112 Sorafenib; Bevacizumab	0.000130406	None	Yes
23	DB00741; DB00860 Hydrocortisone; Prednisolone	0.000130406	None	No
24	DB01132; DB00412 Pioglitazone; Rosiglitazone	0.000130406	PPARG	No
25	DB00480; DB00987 Lenalidomide; Cytarabine	0.000130406	None	No
26	DB00398; DB00002 Sorafenib; Cetuximab	0.000130406	None	No
27	DB00564; DB00776 Carbamazepine; Oxcarbazepine	0.000130406	SCN5A	No
34	DB00762; DB01101 Irinotecan; Capecitabine	0.000260813	None	Yes
35	DB00480; DB01041 Lenalidomide; Thalidomide	0.000260813	PTGS2	Yes

Novel predictions (not reported in DCDB) in the top 35 ranked drug combinations, where only drugs having at least 2 neighbors were included, resulting in 74 positive drug combinations and 1107 random ones.

If we only considered the combinations whose drug components have at least 3 neighbors, termed as DCPred3 (the blue curve in Figure 6), we predicted 40 combinations and 379 negative ones (Additional file 4). DCPred3 achieves an AUC score of 0.92. Compared with the aforementioned two models DCPred1 and DCPred2, based on the information of at least 3 neighor drugs, DCPred3 leads to the overall best performance. In this work, we considered the results by DCPred2 as the final results because only few drugs have more than two neighbors in the drug cocktail network. We hope that the DCPred models developed in this study can be used to facilitate the *in silico *identification of effective drug combinations and speed up the future discovery process.

## Conclusions

Drug combination is a promising strategy for combating complex disease, but our complete understanding of the underlying mechanisms of drug combination is largely lacking at present. It is therefore imperative to develop efficient computational methods to infer effective drug combinations in order to reduce the labor-intensive, time consuming trial-and-error experiments. In this article, we extracted all the known effective drug combinations from DCDB and constructed a drug cocktail network, which includes 215 drugs and 239 effective drug combinations. Based on this cocktail network, we observed that the star drugs tend to have therapeutic similarity with their drug neighbors, and two drugs having similar therapy and sharing neighbors tend to be employed in drug combination. Our analysis also revealed that: 1) hub drugs usually have similar and even the same therapeutic effects as their neighbors; 2) target proteins of the hub drugs are often membrane or membrane-associated proteins; 3) the components in effective drug combinations usually have more similar therapeutic effects, making the drug cocktail network significantly different from the random combination networks.

From the above observations, we consequently developed a new statistical approach to infer and rank possible effective drug combinations by taking into account drugs with at least two or three drug neighbors. As a result, our DCPred2 and DCPred3 models achieved the AUC scores of 0.88 and 0.92, respectively, demonstrating a good performance. We further applied these models to rank all the possible drug combinations and found that the top ranked combinations are more likely to be effective combinations, according to the cross-reference to the literature or the similarity of their ATC codes. In particular, four combinations in the top 35 rankings have been verified as effective combinations by the literature search. We also show that there is a better chance for another 3 combinations to be effective combinations in terms of the pharmacological similarity. Our results in this study provide useful insights into the underlying mechanisms of effective drug combinations and hence important clues for efficiently reducing the search space of possible combinations within the approved drugs. Our approach may be further useful for developing more accurate models. The DCPred models are anticipated to be applied to screen more effective drug combinations with clinical importance.

Furthermore, the concentration of each drug in a combination is a crucial factor in the study of drug combination. However, it is currently difficult to utilize the dosage information of drugs without the knowledge of their quantitative dose-response profiles (e.g. drug induced gene/protein expression data) under different drug concentrations, due to the limited availability of such data. We will investigate drug combinations from this perspective in the future, when more data regarding drug concentrations become available.

## Methods

### Data sources

The annotations of drug combinations were retrieved from a newly released Drug Combination Database (DCDB) [21]. This is a major resource for collecting effective drug combinations from the literature. The target protein information, the Anatomical Therapeutic Chemical (ATC) code annotation of the drugs and protein subcellular localizations, were extracted from DrugBank [22]. Drug combinations that do not have ATC codes for the corresponding drug components and combinations with none or unclear efficacy were discarded. Finally, 194 effective drug combinations were obtained, including 76 approved combinations, 64 clinical combinations and 54 preclinical combinations. We then split the combinations with more than two drug components into combination pairs, resulting in 239 drug combination pairs. These drug combinations were used to construct a drug cocktail network (Figure 1), where the nodes represent drugs and the edges represent combinations, respectively. In the drug cocktail network, the size of each node denotes its degree and the width of each edge denotes the therapeutic similarity (*TS*) between the two drugs linked by the edge. The gray edge means that there is no therapeutic similarity between the two drugs.

Human protein-protein interactions (PPIs) with high confidence from STRING [30] were used to annotate this drug cocktail network, which includes 169,603 interactions between 11,289 proteins after removing pairs with low scores ( < 700).

### Drug therapeutic similarity

The Anatomical Therapeutic Chemical (ATC) Classification System, which includes 5 different hierarchical levels, was used to classify drugs into different groups according to the organ they acted on and the therapeutic chemical characteristics. The *k-*th level drug therapeutic similarity (*S_k_*) between two drugs is defined using the ATC codes of these two drugs:

(2) $$S_{k}\left(d_{1},d_{2}\right)=\frac{ATC_{k}\left(d_{1}\right)\cap ATC_{k}\left(d_{2}\right)}{ATC_{k}\left(d_{1}\right)\cup ATC_{k}\left(d_{2}\right)}$$

where *ATC_k_(d) *denotes all the ATC codes at the *k-*th level of drug *d*. Note that a drug has five levels of ATC codes. A score, *TS*, is used to define the therapeutic similarity between two drugs:

(3) $$TS\left(d_{1},d_{2}\right)=\frac{\overset{n}{\underset{k=1}{\sum }}S_{k}\left(d_{1},d_{2}\right)}{n}$$

where *n *ranges from 1 to 5. In this study, *n *= 3 is adopted considering that only a few drugs have the same ATC codes at the 5^th ^level.

### Drug combination prediction

We assume that two drugs are more likely to be combined if they share a large number of common drugs in the drug cocktail network. For example, if two drugs *d_1 _*and *d*_2 _with respective *n*_1 _and *n*_2 _partners have *m *in common in the drug cocktail network, there will be three groups in the neighborhood of the two drugs, i.e. (1) *m *drugs that are the neighbors of both drug *d*_1 _and *d*_2_; (2) *n*_1 _- *m *partners that are the neighbors of drug *d*_1 _only; and (3) *n*_2 _- *m *partners are the neighbors of drug *d*_2 _only [45]. Suppose that there are totally *N *drugs in the drug combination network, then a *p*-value between *d*_1 _and *d*_2 _can be calculated using the following equation:

(4) $$P\left(m,n_{1},n_{2},N\right)=\frac{\left(\begin{array}{c} N\\ m \end{array}\right)\left(\begin{array}{c} N-m\\ n_{1}-m \end{array}\right)\left(\begin{array}{c} N-n_{1}\\ n_{2}-m \end{array}\right)}{\left(\begin{array}{c} N\\ n_{1} \end{array}\right)\left(\begin{array}{c} N\\ n_{2} \end{array}\right)}$$

If two drugs share more common drugs compared with all of their neighbors, the *p*-value computed by equation (4) will be closer to 0, which means they are more likely to be combined. We use the equation (4) to compute the *p*-values for all possible combinations and then rank the values in ascending order. As drug pairs with lower *p*-values are more likely to be combined, the prediction of effective drug combinations can be made given a certain *p*-value threshold. We term this framework that explores the drug cocktail network and predicts possible drug combination as DCPred (Drug Combination Predictor) and assess its performance for inferring effective drug combinations based on the curated drug combinations dataset.

## Competing interests

The authors declare that they have no competing interests.

## Authors' contributions

KJX implemented the computational method, carried out data analysis, and drafted the manuscript under the direction of XMZ and JS. JS and XMZ guided and coordinated the project, and improved the presentation of the manuscript by copy-editing and fixing language issues. All authors read, revised and approved the final manuscript.

## Supplementary Material

Additional file 1 **The annotation of the neighbor drugs of DB00999 (Hydrochlorothiazide)**. Click here for file

Additional file 2 **The annotation of the neighbor drugs of DB00072 (Trastuzumab)**. Click here for file

Additional file 3 **The ranking of 1181 possible combinations by the DCPred2 model**. Click here for file

Additional file 4 **The ranking of 419 possible combinations by the DCPred3 model**. Click here for file

Additional file 5 **Prediction performance of the DCPred1 model at varying thresholds, as measured by Sensitivity, Specificity and Accuracy**. Click here for file

Additional file 6 **Prediction performance of the DCPred2 model at varying thresholds, as measured by Sensitivity, Specificity and Accuracy**. Click here for file

Additional file 7 **Prediction performance of the DCPred3 model at varying thresholds, as measured by Sensitivity, Specificity and Accuracy**. Click here for file

## References

[B1] Argiris A Wang CX Whalen SG DiGiovanna MP Synergistic interactions between tamoxifen and trastuzumab (Herceptin) Clinical Cancer Research 2004 10 1409 1420 10.1158/1078-0432.CCR-1060-02 14977844

[B2] Osborne CK Schiff R Growth factor receptor cross-talk with estrogen receptor as a mechanism for tamoxifen resistance in breast cancer Breast 2003 12 362 367 10.1016/S0960-9776(03)00137-1 14659106

[B3] Marsh JC Bertino JR Katz KH Davis CA Durivage HJ Rome LS Richards F Capizzi RL Farber LR Pasquale DN The influence of drug interval on the effect of methotrexate and fluorouracil in the treatment of advanced colorectal cancer J Clin Oncol 1991 9 371 380 1999706 10.1200/JCO.1991.9.3.371

[B4] Wilson DR Honrath U Sonnenberg H Interaction of amiloride and hydrochlorothiazide with atrial natriuretic factor in the medullary collecting duct Can J Physiol Pharmacol 1988 66 648 654 10.1139/y88-101 2970886

[B5] Frank J Managing hypertension using combination therapy American Family Physician 2008 77 1279 1286 18540493

[B6] Chan R Park KC Lee MH Lee ES Chang SE Leow YH Tay YK Legarda-Montinola F Tsai RY Tsai TH A randomized controlled trial of the efficacy and safety of a fixed triple combination (fluocinolone acetonide 0.01%, hydroquinone 4%, tretinoin 0.05%) compared with hydroquinone 4% cream in Asian patients with moderate to severe melasma Br J Dermatol 2008 159 697 703 18616780 10.1111/j.1365-2133.2008.08717.x

[B7] Agrawal N Pallos J Slepko N Apostol BL Bodai L Chang LW Chiang AS Thompson LM Marsh JL Identification of combinatorial drug regimens for treatment of Huntington's disease using Drosophila Proceedings of the National Academy of Sciences of the United States of America 2005 102 3777 3781 10.1073/pnas.0500055102 15716359 PMC553288

[B8] Campostrini N Marimpietri D Totolo A Mancone C Fimia GM Ponzoni M Righetti PG Proteomic analysis of anti-angiogenic effects by a combined treatment with vinblastine and rapamycin in an endothelial cell line Proteomics 2006 6 4420 4431 10.1002/pmic.200600119 16888724

[B9] Lehar J Krueger AS Avery W Heilbut AM Johansen LM Price ER Rickles RJ Short GF Staunton JE Jin X Synergistic drug combinations tend to improve therapeutically relevant selectivity Nature Biotechnology 2009 27 659 666 10.1038/nbt.1549 PMC2708317 19581876

[B10] Zimmermann GR Lehar J Keith CT Multi-target therapeutics: when the whole is greater than the sum of the parts Drug Discovery Today 2007 12 34 42 10.1016/j.drudis.2006.11.008 17198971

[B11] Pennati M Campbell AJ Curto M Binda M Cheng YZ Wang LZ Curtin N Golding BT Griffin RJ Hardcastle IR Potentiation of paclitaxel-induced apoptosis by the novel cyclin-dependent kinase inhibitor NU6140: a possible role for survivin down-regulation Molecular Cancer Therapeutics 2005 4 1328 1337 10.1158/1535-7163.MCT-05-0022 16170024

[B12] Lewis BR Aoun SL Bernstein GA Crow SJ Pharmacokinetic interactions between cyclosporine and bupropion or methylphenidate Journal of Child and Adolescent Psychopharmacology 2001 11 193 198 10.1089/104454601750284117 11436960

[B13] Tari L Anwar S Liang S Cai J Baral C Discovering drug-drug interactions: a text-mining and reasoning approach based on properties of drug metabolism Bioinformatics 2010 26 i547 553 10.1093/bioinformatics/btq382 20823320 PMC2935409

[B14] Zhao XM Wang RS Chen L Aihara K Uncovering signal transduction networks from high-throughput data by integer linear programming Nucleic Acids Res 2008 36 e48 10.1093/nar/gkn145 18411207 PMC2396433

[B15] Zhao XM Wang RS Chen L Aihara K Automatic modeling of signaling pathways by network flow model J Bioinform Comput Biol 2009 7 309 322 10.1142/S0219720009004138 19340917

[B16] Geva-Zatorsky N Dekel E Cohen AA Danon T Cohen L Alon U Protein Dynamics in Drug Combinations: a Linear Superposition of Individual-Drug Responses Cell 2010 140 643 651 10.1016/j.cell.2010.02.011 20211134

[B17] Calzolari D Bruschi S Coquin L Schofield J Feala JD Reed JC McCulloch AD Paternostro G Search Algorithms as a Framework for the Optimization of Drug Combinations Plos Computational Biology 2008 4 e1000249 10.1371/journal.pcbi.1000249 19112483 PMC2590660

[B18] Jin G Zhao H Zhou X Wong ST An enhanced Petri-net model to predict synergistic effects of pairwise drug combinations from gene microarray data Bioinformatics 2011 27 i310 i316 10.1093/bioinformatics/btr202 21685086 PMC3117391

[B19] Wu Z Zhao XM Chen L A systems biology approach to identify effective cocktail drugs BMC Syst Biol 2010 4 Suppl 2 S7 10.1186/1752-0509-4-S2-S7 20840734 PMC2982694

[B20] Zhao XM Iskar M Zeller G Kuhn M Noort V Bork P Prediction of drug combinations by integrating molecular and pharmacological data Plos Computational Biology 2011 7 e1002323 10.1371/journal.pcbi.1002323 22219721 PMC3248384

[B21] Liu YB Hu B Fu CX Chen X DCDB: Drug combination database Bioinformatics 2010 26 587 588 10.1093/bioinformatics/btp697 20031966

[B22] Wishart DS Knox C Guo AC Cheng D Shrivastava S Tzur D Gautam B Hassanali M DrugBank: a knowledgebase for drugs, drug actions and drug targets Nucleic Acids Research 2008 36 D901 D906 18048412 10.1093/nar/gkm958 PMC2238889

[B23] Newman MEJ The structure and function of complex networks Siam Review 2003 45 167 256 10.1137/S003614450342480

[B24] Maslov S Sneppen K Specificity and stability in topology of protein networks Science 2002 296 910 913 10.1126/science.1065103 11988575

[B25] Barabasi AL Oltvai ZN Network biology: understanding the cell's functional organization Nature Reviews Genetics 2004 5 101 113 10.1038/nrg1272 14735121

[B26] MacKay JH Arcuri KE Goldberg AI Snapinn SM Sweet CS Losartan and low-dose hydrochlorothiazide in patients with essential hypertension - A double-blind, placebo-controlled trial of concomitant administration compared with individual components Archives of Internal Medicine 1996 156 278 285 10.1001/archinte.1996.00440030072009 8572837

[B27] Salmela PI Juustila H Kinnunen O Koistinen P Comparison of low doses of hydrochlorothiazide plus amiloride and hydrochlorothiazide alone in hypertension in elderly patients Ann Clin Res 1986 18 88 92 3521443

[B28] Romond EH Perez EA Bryant J Suman VJ Geyer CE Davidson NE Tan-Chiu E Martino S Paik S Kaufman PA Trastuzumab plus adjuvant chemotherapy for operable HER2-positive breast cancer New England Journal of Medicine 2005 353 1673 1684 10.1056/NEJMoa052122 16236738

[B29] Piccart-Gebhart MJ Procter M Leyland-Jones B Goldhirsch A Untch M Smith I Gianni L Baselga J Bell R Jackisch C Trastuzumab after adjuvant chemotherapy in HER2-positive breast cancer New England Journal of Medicine 2005 353 1659 1672 10.1056/NEJMoa052306 16236737

[B30] Jensen LJ Kuhn M Stark M Chaffron S Creevey C Muller J Doerks T Julien P Roth A Simonovic M STRING 8-a global view on proteins and their functional interactions in 630 organisms Nucleic Acids Research 2009 37 D412 D416 10.1093/nar/gkn760 18940858 PMC2686466

[B31] Hand D Measuring classifier performance: a coherent alternative to the area under the ROC curve Machine Learning 2009 77 103 123 10.1007/s10994-009-5119-5

[B32] Fawcett T An introduction to ROC analysis Pattern Recognition Letters 2006 27 861 874 10.1016/j.patrec.2005.10.010

[B33] Delord JP Pierga JY Dieras V Bertheault-Cvitkovic F Turpin FL Lokiec F Lochon I Chatelut E Canal P Guimbaud R A phase I clinical and pharmacokinetic study of capecitabine (Xeloda[reg]) and irinotecan combination therapy (XELIRI) in patients with metastatic gastrointestinal tumours Br J Cancer 2005 92 820 826 10.1038/sj.bjc.6602354 15756252 PMC2361914

[B34] Garcia-Alfonso P Munoz-Martin A Mendez-Urena M Quiben-Pereira R Gonzalez-Flores E Perez-Manga G Capecitabine in combination with irinotecan (XELIRI), administered as a 2-weekly schedule, as first-line chemotherapy for patients with metastatic colorectal cancer: a phase II study of the Spanish GOTI group Br J Cancer 2009 101 1039 1043 10.1038/sj.bjc.6605261 19738605 PMC2768107

[B35] Dent S Messersmith H Trudeau M Gemcitabine in the management of metastatic breast cancer: a systematic review Breast Cancer Research and Treatment 2008 108 319 331 10.1007/s10549-007-9610-z 17530427

[B36] Chang J Makris A Gutierrez M Hilsenbeck S Hackett J Jeong J Liu M-L Baker J Clark-Langone K Baehner F Gene expression patterns in formalin-fixed, paraffin-embedded core biopsies predict docetaxel chemosensitivity in breast cancer patients Breast Cancer Research and Treatment 2008 108 233 240 10.1007/s10549-007-9590-z 17468949

[B37] Levy C Fumoleau P Gemcitabine plus docetaxel: a new treatment option for anthracycline pretreated metastatic breast cancer patients? Cancer Treatment Reviews 2005 31 S17 S22 16360543 10.1016/s0305-7372(05)80004-0

[B38] Wilhelm SM Adnane L Newell P Villanueva A Llovet JM Lynch M Preclinical overview of sorafenib, a multikinase inhibitor that targets both Raf and VEGF and PDGF receptor tyrosine kinase signaling Molecular Cancer Therapeutics 2008 7 3129 3140 10.1158/1535-7163.MCT-08-0013 18852116 PMC12261297

[B39] Los M Roodhart JML Voest EE Target practice: Lessons from phase III trials with bevacizumab and vatalanib in the treatment of advanced colorectal cancer Oncologist 2007 12 443 450 10.1634/theoncologist.12-4-443 17470687

[B40] Azad NS Posadas EM Kwitkowski VE Steinberg SM Jain L Annunziata CM Minasian L Sarosy G Kotz HL Premkumar A Combination targeted therapy with sorafenib and bevacizumab results in enhanced toxicity and antitumor activity Journal of Clinical Oncology 2008 26 3709 3714 10.1200/JCO.2007.10.8332 18669456 PMC9089757

[B41] Kumar S Rajkumar SV Thalidomide and lenalidomide in the treatment of multiple myeloma European Journal of Cancer 2006 42 1612 1622 10.1016/j.ejca.2006.04.004 16750621

[B42] Anderson KC Lenalidomide and thalidomide: Mechanisms of action - Similarities and differences Seminars in Hematology 2005 42 S3 S8 16344099 10.1053/j.seminhematol.2005.10.001

[B43] Horrobin DF A low toxicity maintenance regime, using eicosapentaenoic acid and readily available drugs, for mantle cell lymphoma and other malignancies with excess cyclin D1 levels Medical Hypotheses 2003 60 615 623 10.1016/S0306-9877(03)00075-6 12710892

[B44] Vallet S Palumbo A Raje N Boccadoro M Anderson KC Thalidomide and lenalidomide: Mechanism-based potential drug combinations Leuk Lymphoma 2008 49 1238 1245 10.1080/10428190802005191 18452080

[B45] Samanta MP Liang S Predicting protein functions from redundancies in large-scale protein interaction networks Proceedings of the National Academy of Sciences of the United States of America 2003 100 12579 12583 10.1073/pnas.2132527100 14566057 PMC240660

