# Supplementary material for: The drug cocktail network
Source: BMC Syst Biol. 2012 Jul 16;6(Suppl 1):S5. doi: 10.1186/1752-0509-6-S1-S5 (PMC3403482; doi:10.1186/1752-0509-6-S1-S5)
Supplement: Additional file 1 — The annotation of the neighbor drugs of DB00999 (Hydrochlorothiazide). [file 1752-0509-6-S1-S5-S1.doc]

Additional file 1

1. Name: Hydrochlorothiazide (DB00999)
2. Description: For the treatment of high blood pressure and management of edema.
3. Combination neighbors: (The first column represents the serial number of the combination including DB00999, the 2^nd^ and 3^rd^ columns represent the pharmacological information of another drug component in the combination, and the others denote the pharmacological information of the drug combination.)

|  | Another drug component | Disease | Combination name id | Disease | Action type | Possible mechanism |
| --- | --- | --- | --- | --- | --- | --- |
| 1 | Amiloride  (DB00594) | hypertension, heart failure | Moduretic  (DC00106) | Hypertension  Hypertension  Hypertension | Different targets of related pathways  Different targets of related pathways | Amiloride inhibites sodium reabsorption through sodium channels in renal epithelial cells, and is used in conjunction with diuretics to spare potassium loss. |
| 2 | Fosinopril  (DB00492) | hypertension, heart failure | Monopril-HCT  (DC00103) |  |  | Fosinopril sodium, is an ACE (angiotensin-converting enzyme) inhibitor. |
| 3 | **Lisinopril**  **(**DB00722**)** | hypertension,  congestive heart failure | Prinzide  (DC00113) |  |  | Lisinopril, the ACE inhibitor, works by limiting production of a substance that promotes salt and water retention in body. |
| **4** | **Losartan**  **(**DB00678**)** | Hypertension | Hyzaar  (DC00114) |  |  | Losartan keeps blood vessels from narrowing, which lowers blood pressure improves blood flow and also tends to reverse the potassium loss associated with diuretics. |
| **5** | **Quinapril**  **(**DB00881**)** | hypertension, chronic heart failure. | Accuretic  (DC00119) |  |  | Quinapril is in a group of drugs called ACE inhibitors. ACE stands for angiotensin converting enzyme. Quinapril lowers blood pressure and also relieves symptoms of fluid retention. |
| **6** | **Spironolactone**  **(**DB00421**)** | low-renin hypertension, hypokalemia, Conn's syndrome. | Aldactazide  (DC00120) |  |  | Spironolactone is a potassium-sparing diuretic that also prevents body from absorbing too much salt and keeps your potassium levels from getting too low. |
| **7** | **Telmisartan**  **(**DB00966**)** | Hypertension | Micardis HCT  (DC00121) |  |  | Telmisartan is in a group of drugs called angiotensin II receptor antagonists. Telmisartan keeps blood vessels from narrowing, which lowers blood pressure and improves blood flow. |
| **8** | **Triamterene**  **(**DB00384**)** | edema associated with congestive heart failure | Dyazide  (DC00122) |  |  | Triamterene is a potassium-sparing diuretic that also prevents body from absorbing too much salt and keeps the potassium level from getting too low. |
| **9** | **Enalapril**  **(**DB00584**)** | Hypertension, heart failure | Vaseretic  (DC00126) |  |  | Enalapril, the ACE inhibitor, works by decreasing salt and water retention in body. Enalapril also enhances blood flow throughout blood vessels. |
| **10** | **Candesartan**  **(**DB00796**)** | Hypertension | Atacand HCT  (DC00128) |  |  | Candesartan blocks the vasoconstrictor and aldosterone-secreting effects. |
| **11** | **Captopril**  **(**DB01197**)** | Hypertension | Capozide  (DC00129) |  |  | Captopril lowers blood pressure and also relieves symptoms of fluid retention. |
| **12** | Irbesartan  (DB01029) | Hypertension, type 2 diabetes | Avalide  (DC00112) |  | Different targets of unrelated pathways  Different targets of related pathways | Irbesartan is in a group of drugs called angiotensin II receptor antagonists which keep blood vessels from narrowing, which lowers blood pressure and improves blood flow. |
| 13 | **Methyldopa**  **(**DB00968**)** | Hypertension | Aldoril  (DC00115) |  |  | Methyldopa works by controlling nerve impulses along certain nerve pathways. As a result, it relaxes blood vessels so that blood passes through them more easily. |
| **14** | **Metoprolol**  **(**DB00264**)** | Hypertension, angina pectoris | Lopressor  (DC00116) |  |  | Metoprolol is in a group of drugs called beta-blockers. Beta-blockers affect the heart and circulation (blood flow through arteries and veins) |
| **15** | **Olmesartan**  **(**DB00275**)** | hypertension | Benicar  (DC00117) |  |  | Olmesartan is in a group of drugs called angiotensin II receptor antagonists. Olmesartan keeps blood vessels from narrowing, which lowers blood pressure and improves blood flow. |
| **16** | **Propranolol**  **(**DB00571**)** | Hypertension, prophylaxis of migraine | Inderide  (DC00118) |  |  | Propranolol is in a group of drugs called beta-blockers. Beta-blockers affect the heart and circulation (blood flow through arteries and veins). |
| **17** | **Valsartan**  **(**DB00177**)** | hypertension | Diovan HCT  (DC00123) |  |  | Valsartan is in a group of drugs called angiotensin II receptor antagonists. Valsartan keeps blood vessels from narrowing, which lowers blood pressure and improves blood flow. |
| **18** | **Bisoprolol**  **(**DB00612**)** | Hypertension, prophylaxis treatment of angina | Ziac  (DC00131) |  |  | Bisoprolol produces a reduction of heart rate, cardiac output, systolic and diastolic blood pressure, and possibly relax orthostatic hypotension. |
