# Supplementary material for: The drug cocktail network
Source: BMC Syst Biol. 2012 Jul 16;6(Suppl 1):S5. doi: 10.1186/1752-0509-6-S1-S5 (PMC3403482; doi:10.1186/1752-0509-6-S1-S5)
Supplement: Additional file 2 — The annotation of the neighbor drugs of DB00072 (Trastuzumab). [file 1752-0509-6-S1-S5-S2.doc]

Additional file **2**

1. **Name: Trastuzumab (**DB00072**)**
2. **Description:** For treatment of HER2-positive metatsatic breast cancer
3. Combination neighbors: (The first column represents the serial number of the combination including DB00072, the 2^nd^ and 3^rd^ columns represent the pharmacological information of another drug component in the combination, and the others denote the pharmacological information of the drug combination.)

|  | another drug component | Disease | Combination name id | diease | Action type | Possible mechanism |
| --- | --- | --- | --- | --- | --- | --- |
| 1 | **Tamoxifen**  **(DB00675)** | breast cancer | MDC1-13  (DC00188) | Breast cancer | Different targets of related pathways | ER crosstalks with EGFR and HER-2/neu, signaling via EGFR and HER-2/neu can activate ER and its coactivator AIB1, ER of cell membrane can activate EGFR/HER-2. Anti-HER-2/neu antibody trastuzumab stopped HER-2/neu induced activation of ER and AIB1. ER antagonist tamoxifen stopped ER induced activation of EGFR/HER-2. Use of both drugs reduced the counteractive crosstalks. |
| 2 | **Bortezomib**  (DB00188) | multiple myeloma | MDC2-4  (DC00200) | Breast cancer | Different targets of the related pathways | Bortezomib's inhibition of NFkappaB is complemented by herceptin's inhibition of HER-2 receptor that subsequently blocks EGF-induced NFkappaB activation. |
| 3 | **Paclitaxel**  (DB01229) | Kaposi's sarcoma and cancer of the lung, ovarian, and breast. | NDC029  (DC00297) | Epidermal growth factor receptor 2(HER2)-positive advanced breast cancer | Different targets of unrelated pathways | Trastuzumab is a monoclonal antibody that interferes with the HER2/neu receptor causing cell cycle arrest. Paclitaxel binds to tubulin and inhibits the disassembly of microtubules, thereby resulting in the inhibition of cell division. This agent also induces apoptosis by binding to and blocking the function of the apoptosis inhibitor protein Bcl-2 (B-cell Leukemia 2). |
| **4** | **Carboplatin**  (DB00958) | possesses antineoplastic activity | NDC194  (DC00462) | HER2-overexpressing breast cancer | Different targets of related pathways | After binding to HER2 on the tumor cell surface, trastuzumab induces an antibody-dependent cell-mediated cytotoxicity against tumor cells that overexpress HER2. Carboplatin contains a platinum atom complexed with two ammonia groups and a cyclobutane-dicarboxyl residue. This agent is activated intracellularly to form reactive platinum complexes that bind to nucleophilic groups such as GC-rich sites in DNA, thereby inducing intrastrand and interstrand DNA cross-links, as well as DNA-protein cross-links. These carboplatin-induced DNA and protein effects result in apoptosis and cell growth inhibition. |
| **5** | **Vinorelbine**  (DB00361) | For the treatment of non-small-cell lung carcinoma | NDC196  (DC00464) | HER2-overexpressing breast cancer | Different targets of related pathways | After binding to HER2 on the tumor cell surface, trastuzumab induces an antibody-dependent cell-mediated cytotoxicity against tumor cells that overexpress HER2. Vinorelbine binds to tubulin, thereby inhibiting tubulin polymerization into microtubules and spindle formation and resulting in apoptosis of susceptible cancer cells. Inhibition of mitotic microtubules correlates with antitumor activity, whereas inhibition of axonal microtubules seems to correlate with vinorelbine's neurotoxicity. |
| **6** | **Doxorubicin**  (DB00997) | Koposi's sarcome connected to AIDS. | NDC197  (DC00465) | HER2-overexpressing breast cancer | Different targets of related pathways | After binding to HER2 on the tumor cell surface, trastuzumab induces an antibody-dependent cell-mediated cytotoxicity against tumor cells that overexpress HER2. Doxorubicin intercalates between base pairs in the DNA helix, thereby preventing DNA replication and ultimately inhibiting protein synthesis. Additionally, doxorubicin inhibits topoisomerase II which results in an increased and stabilized cleavable enzyme-DNA linked complex during DNA replication and subsequently prevents the ligation of the nucleotide strand after double-strand breakage. |
| **7** | **Epirubicin**  (DB00445) | axillary node tumor involvement following resection of primary breast cancer | NDC198  (DC00466) | HER2-overexpressing breast cancer | Different targets of related pathways | After binding to HER2 on the tumor cell surface, trastuzumab induces an antibody-dependent cell-mediated cytotoxicity against tumor cells that overexpress HER2. Epirubicin intercalates into DNA and interacts with topoisomerase II, thereby inhibiting DNA replication and repair and RNA and protein synthesis. |
| **8** | **Gemcitabine**  (DB00441) | metastatic breast cancer | NDC199  (DC00467) | HER2-overexpressing breast cancer | Different targets of related pathways | After binding to HER2 on the tumor cell surface, trastuzumab induces an antibody-dependent cell-mediated cytotoxicity against tumor cells that overexpress HER2. Gemcitabine is converted intracellularly to the active metabolites difluorodeoxycytidine di- and triphosphate (dFdCDP, dFdCTP). dFdCDP inhibits ribonucleotide reductase, thereby decreasing the deoxynucleotide pool available for DNA synthesis; dFdCTP is incorporated into DNA, resulting in DNA strand termination and apoptosis. |
| **9** | **Docetaxel**  (DB01248) | metastatic breast cancer | NDC200  (DC00468) | HER2-overexpressing breast cancer | Different targets of related pathways | Docetaxel displays potent and broad antineoplastic properties; it binds to and stabilizes tubulin, thereby inhibiting microtubule disassembly which results in cell- cycle arrest at the G2/M phase and cell death. This agent also inhibits pro-angiogenic factors such as vascular endothelial growth factor (VEGF) and displays immunomodulatory and pro-inflammatory properties by inducing various mediators of the inflammatory response. After binding to HER2 on the tumor cell surface, trastuzumab induces an antibody-dependent cell-mediated cytotoxicity against tumor cells that overexpress HER2. Carboplatin contains a platinum atom complexed with two ammonia groups and a cyclobutane-dicarboxyl residue. This agent is activated intracellularly to form reactive platinum complexes that bind to nucleophilic groups such as GC-rich sites in DNA, thereby inducing intrastrand and interstrand DNA cross-links, as well as DNA-protein cross-links. These carboplatin-induced DNA and protein effects result in apoptosis and cell growth inhibition. |
| **10** | **Cisplatin**  **(DB00515)** | metastatic testicular tumors, metastatic ovarian tumors and advanced bladder cancer | MDC1-18  (DC00193) | Gastric cancer | Different targets of crosstalking pathways | Cisplatin formed DNA adduct to induce DNA damage and apoptosis, which may be attenuated by DNA repair systems in certain cell types. This counteractive DNA repair action may be partially reduced by herceptin's anti-HER2 activitity that suppressed DNA repair pathway known to crosstalk to HER2 and inhibited PI3KAKT pathway to enhance apoptosis. |
