# Supplementary material for: The drug cocktail network
Source: BMC Syst Biol. 2012 Jul 16;6(Suppl 1):S5. doi: 10.1186/1752-0509-6-S1-S5 (PMC3403482; doi:10.1186/1752-0509-6-S1-S5)
Supplement: Additional file 5 — Prediction performance of the DCPred1 model at varying thresholds, as measured by Sensitivity, Specificity and Accuracy. [file 1752-0509-6-S1-S5-S5.doc]

Additional file 5. Prediction performance of DCPred1 model at varying thresholds, as measured by Sensitivity, Specificity and Accuracy.

| Threshold | Sensitivity | Specificity | Accuracy |
| --- | --- | --- | --- |
| 100 | 0.3378 | 0.9322 | 0.8950 |
| 200 | 0.7973 | 0.8726 | 0.8679 |
| 300 | 0.7973 | 0.7823 | 0.7832 |
| 400 | 0.7973 | 0.6920 | 0.6986 |
| 500 | 0.8784 | 0.6070 | 0.6240 |

Note that the threshold was applied in a way such that those combinations ranked above it were considered as positive combinations.
