# Supplementary material for: The drug cocktail network
Source: BMC Syst Biol. 2012 Jul 16;6(Suppl 1):S5. doi: 10.1186/1752-0509-6-S1-S5 (PMC3403482; doi:10.1186/1752-0509-6-S1-S5)
Supplement: Additional file 6 — Prediction performance of the DCPred2 model at varying thresholds, as measured by Sensitivity, Specificity and Accuracy. [file 1752-0509-6-S1-S5-S6.doc]

Additional file 6. Prediction performance of DCPred2 model at varying thresholds, as measured by Sensitivity, Specificity and Accuracy.

| Threshold | Sensitivity | Specificity | Accuracy |
| --- | --- | --- | --- |
| 100 | 0.5811 | 0.9485 | 0.9255 |
| 200 | 0.8108 | 0.8735 | 0.8696 |
| 300 | 0.8919 | 0.7886 | 0.7951 |
| 400 | 0.9054 | 0.6992 | 0.7121 |
| 500 | 0.9054 | 0.6089 | 0.6274 |

Note that the threshold was applied in a way such that those combinations ranked above it were considered as positive combinations.
