# Supplementary material for: The drug cocktail network
Source: BMC Syst Biol. 2012 Jul 16;6(Suppl 1):S5. doi: 10.1186/1752-0509-6-S1-S5 (PMC3403482; doi:10.1186/1752-0509-6-S1-S5)
Supplement: Additional file 7 — Prediction performance of the DCPred3 model at varying thresholds, as measured by Sensitivity, Specificity and Accuracy. [file 1752-0509-6-S1-S5-S7.doc]

Additional file 7. Prediction performance of DCPred3 model at varying thresholds, as measured by Sensitivity, Specificity and Accuracy.

| Threshold | Sensitivity | Specificity | Accuracy |
| --- | --- | --- | --- |
| 100 | 0.8500 | 0.9422 | 0.9390 |
| 200 | 0.9750 | 0.8589 | 0.8628 |
| 300 | 1 | 0.7721 | 0.7798 |
| 400 | 1 | 0.6845 | 0.6952 |
| 500 | 1 | 0.6678 | 0.6791 |

Note that the threshold was applied in a way such that those combinations ranked above it were considered as positive combinations.
